# Reporting preclinical gene therapy studies in the field of Niemann-Pick type C disease according to the ARRIVE guidelines

**DOI:** 10.1186/s13023-024-03479-1

**Published:** 2025-05-06

**Authors:** Charlotte Laurfelt Munch Rasmussen, Annette Burkhart, Torben Moos, Louiza Bohn Thomsen

**Affiliations:** 1 Neurobiology Research and Drug Delivery, Department of Health Science and Technology, Aalborg University, Selma Lagerløfs Vej 249, DK-9260 Gistrup, Denmark; 2 The Biomedical Laboratory, Department of Molecular Medicine, University of Southern Denmark, J.B. Winsløws Vej 23, DK-5000 Odense C, Denmark

**Keywords:** Niemann-Pick type C disease, NPC, Gene therapy, ARRIVE guidelines, ARRIVE 2.0

## Abstract

The lack of essential information when reporting animal studies causing lower reproducibility has been stressed for decades. The ARRIVE (Animal Research: Reporting of In Vivo Experiments) guidelines were first published in 2010, to improve reporting of animal research, making in vivo studies more transparent thereby improving the scientific quality. Regardless of an endorsement from the scientific community, there is still a continuous need to improve animal research reporting, which unfortunately also is the case in the field of Niemann-Pick type C disease (NPC). NPC is a lipid storage disorder, caused by mutations in either the *Npc1* or *Npc2* gene. Despite years of research, no cure for this fatal disease exists. In 2020, an updated version of the ARRIVE guidelines (ARRIVE 2.0), was published, describing the ten most essential elements to be included when reporting pre-clinical studies. Here we systematically reviewed the compliance with the ARRIVE guidelines using the “ARRIVE Essential 10” checklist in a series of pre-clinical studies investigating gene therapy as a treatment strategy for NPC. None of the reviewed papers fulfilled the ARRIVE 2.0 guidelines. Information regarding sample size, randomization, blinding, and statistical methodology was lacking. Hopefully, the newly updated ARRIVE guidelines will aid researchers in planning and publishing in vivo experiments in the future. More awareness of the importance of including these essential items is needed, both from editors, reviewers and researchers, for complete endorsement of the ARRIVE guidelines in the scientific community.

## Introduction

Niemann-Pick type C (NPC) disease is a severe autosomal recessive, neurovisceral disorder characterized by the accumulation of cholesterol and other lipids in lysosomes [1]. The etiology of NPC disease resides in loss-of-function mutations in one of the two genes, *Npc1* (95% of the cases) or *Npc2* (5% of the cases), resulting in impaired cholesterol transport out of the lysosomes, subsequently altering the lipid metabolism, which is a critical event in the pathogenesis [2–4]. The progressive nature of the early onset of symptoms, including ataxia, dysphagia, dystonia, and dementia, leads to premature death [5].

Unfortunately, there is no cure for NPC disease and to date, Miglustat is the only approved therapeutic option used for decelerating disease progression by inhibiting the synthesis of glycosphingolipids [6–8]. Therefore, the development of new treatment strategies is needed, but due to the blood–brain barrier, the delivery of drugs, proteins, or genes to the brain is hindered [9]. Within the last years, gene therapy has been explored as a potential option for the treatment of NPC disease. Several studies using NPC-deficient (*Npc*-/-) mouse models have demonstrated improvements in survival and ameliorating brain pathology after the administration of viral vectors [10–16]. Animal models for studying disease mechanisms and therapeutic effects, e.g., gene therapy, are of great importance for NPC disease, but an ongoing discussion related to animal models, is that we are in a translational and reproducibility crisis, and there may be several possible explanations for this [17].

In June 2010, the “Animal Research: Reporting of In Vivo Experiments (ARRIVE) guidelines” were published to improve the quality of reporting animal studies in scientific publications [18]. Sufficient information should be reported allowing for a constructive review of animal studies, making the in vivo studies reproducible, and thus preventing unnecessary use of animals [18, 19]. The ARRIVE guidelines provide a checklist describing the minimum information, that should be included when publishing research using animals, e.g., information regarding the experimental animals (number, species, strain, sex, and genetic background), experimental design, and statistical and analytical methods [18]. Ten years later, an update regarding the ARRIVE guidelines was published (ARRIVE 2.0) due to a continuous need for improvement in reporting animal experiments related to the inconsistency in adherence to the original guidelines [20].

Systematic reviews of the literature in a field of research are a valuable tool when designing in vivo studies, identifying research gaps, and subsequently justifying why new animal studies are needed [20]. In designing our gene therapy study in NPC2-deficient mice (unpublished data), a systematic review of gene therapy studies was an essential part of the planning.

The main objective of this review is, therefore, to evaluate studies assessing the efficacy of gene therapy in mouse models of NPC disease according to the “ARRIVE Essential 10”, i.e., the minimum information to be included in a manuscript [20].

## Criteria for inclusion and exclusion of publications

ARRIVE guidelines were first published in June 2010; therefore, only studies published after June 2010 have been included to accommodate the scientific adoption of these new guidelines for publishing animal studies [21]. Both studies using non-viral and viral gene therapy were included with the viral gene therapy studies being limited to adeno-associated virus (AAV), which is the most widely used vector in mouse models of NPC disease [11]. Only data obtained from the treatment studies were included and study design and results regarding gene expression analysis in wild-type mice were excluded. The main focus of this review is on the information essential to assessing the reliability of the presented findings, “ARRIVE Essential 10”, which includes details on the study design, sample size, measures to reduce subjective bias, outcome measures, statistical methods, the animals, experimental procedures, and results [20]. With these inclusion criteria stated, eight articles were included. An overview of these studies can be seen in Table 1.

**Table 1:** Overview of the included studies

Mouse model	Vector	Promoter	Dosage per mouse	Route of administration	Age at treatment	Effect		Reference
						CNS	P	
Viral gene therapy								
*Npc1*^*nih/nih*^ *Npc1*^*nmf164*^	AAV9	GAPDH PGK NPC1 CBA hSYN1 SYN-S SYN-D CAG	1.5 × 10^11^ vg	Intracerebroventricular injection	PN 0–1	+	n.a.	[16]
*BALB/cNctr-Npc1*^*m1N*^*/J*	AAV9 and AAV-PHP.B	EF1α	1.84 × 10^12^ vg AAV9 1.43 × 10^12^ vg AAV-PHP.B	Retro-orbital injection	PN 24–27	+	+	[11]
*FVB.C-Npc1*^*m1N*^*/J*	AAV9/3	CMV	1.35 × 10^11^ vg	Intracerebroventricular and intracisternal injections	PN 4–5	+	+	[12]
*BALB/cNctr-Npc1*^*m1N*^*/J*	AAV9	hSYN1	4.6 × 10^9^ vg or 2.5 × 10^11^ vg	Bilateral intra-cerebroventricular injections	PN 0–1	+	−	[15]
*Npc2*^*tm1Plob*^	AAVrh.10	CAG	10^11^ vg	Intracisternal injection	6 weeks	+	+	[14]
*BALB/cNctr-Npc1*^*m1N*^*/J*	AAV9	CMV	2.5 × 10^11^ vg	Intracardiac injection	PN 4	+	+	[13]
*BALB/cNctr-Npc1*^*m1N*^*/J*	AAV9	CamKII or EF1α	2.6 × 10^11^ vg (neonates) 1.3 × 10^12^ vg (juvenile)	Retro-orbital injection	PN 1–3 PN 20–25	+	+	[10]
Non-viral gene therapy								
*BALB/cNctr-Npc1*^*m1N*^*/J*	TfRMAb targeted THLs	PDGFB	6 µg plasmid DNA and 15 µg TfRMAb	Tail-vein injection	6–7 weeks	−	+	[22]

*AAV* Adeno-associated virus, *GAPDH* glyceraldehyde-3-phosphate dehydrogenase, *PGK* phosphoglycerate kinase, *NPC1* small, truncated version of the endogenous human NPC1 promoter, *CBA* chicken β-actin, *hSYN1* human synapsin 1, *SYN-D* neuron de-targeted synapsin-1, *SYN-S* shortened synapsin-1, *CAG* chicken β-actin promoter with CMV enhancer, EF1α: elongation factor 1α, *CMV* cytomegalovirus, CamKII: calcium/calmodulin-dependent protein kinase II, TfrMAb targeted *THLs* Transferrin receptor-specific monoclonal antibody conjugated to *THLs* Trojan horse liposomes, *PDGFB* human platelet-derived growth factor B, *vg* viral genomes, *PN* postnatal day, *CNS* central nervous system, *P* peripheral, *n.a*. not assessed

With this in mind, the selected publications' compliance with the ARRIVE guidelines was evaluated, focusing on the “Material and Methods” section. Each of the 10 points will be discussed separately, highlighting the importance of including these specific items in a manuscript. Importantly, the intention was not to question the results reported in the included papers, nor to criticize the experimental work performed by the authors, but to provide more awareness of the ARRIVE guidelines [21]. In addition, this review can also provide an overview of the studies in the field of NPC in terms of different vectors used, doses, etc. Subsequently, it will be helpful in the planning of future animal studies using NPC disease models.

## Study design

According to the ARRIVE guidelines, the minimum information included when reporting study design is details of the different groups being compared as well as the experimental unit. All studies have used a case–control design, where the *Npc-/-* mice are allocated to a control or treatment group. Most of the studies also include a group of untreated wild-type mice. Even though the study design is comparable, the *Npc-/-* control group differs. In four out of eight studies, the control group consisted of vehicle (phosphate-buffered saline (PBS), HEPES buffer, or saline) treated *Npc-/-* mice [10–12, 22], whereas in two other studies, no information about the *Npc-/-* control group is available [14, 15]. One study includes untreated *Npc-/-* mice [16]. Two studies have used a control vector expressing a reporter construct, green fluorescent protein (GFP), instead of the protein of interest [10, 13]. One of the aforementioned studies includes both a PBS and an AAV-GFP-treated *Npc-/-* control group [10]. None of the studies report how the *Npc-/-* mice are allocated to the different experimental groups. Only one study provides details of the experimental unit [11].

## Sample size

The following information about the sample size should be included; 1) the total number of animals used, 2) the total number in each group, and 3) the total number in each experiment, and finally, 4) how the sample size was decided should be explained [20]. Especially the last part, i.e., number four, is very important concerning the 3Rs, reduction, refinement, and replacement. The sample size should be sufficient to detect the intended effect without using more animals than necessary. A sample size calculation can accommodate this issue, ensuring that the study is neither underpowered nor overpowered [23]. Even though this information is an essential part of reporting animal studies, only two studies clearly state the total number of animals used [11, 22] (Table 2). For additional specifications of experimental groups, see Table 3. Only one study substantiated how the sample size was determined [11].

**Table 2:** Study design

References	Sample size	Group size			Randomization/ Blinding	Study period*	Statistics^**^
		WT	CTRL	T			
[16]	n.s. [66]	6	6	54	−/−	10 weeks	−
[11]	55	13	13	29	+ / +	9 weeks	+
[12]	n.s. [46]	11	23	12	−/−	11 weeks	−
[22]	36	n.s.	18	18	−/−	5 weeks	N
[15]	n.s. [38]	6	8	24	−/−	Up to 17 weeks^#^	−
[14]	n.s.	n.s.	n.s.	n.s.	−/−	10 weeks	N
[13]	n.s.	n.s.	n.s.	n.s.	−/−	8 weeks	−
[10]	n.s. [44]	0	22	22	−/ +	9 weeks	−

*WT* wild-type, *CTRL* untreated *Npc*-/- mice, *T* treated *Npc*-/- mice, *refer to the duration of the study used for immunohistochemical evaluation (from injection to euthanasia), **fulfillment of the criteria stated in the ARRIVE guidelines, n.s. = not specified, e.g. the number of animals is not stated clearly in the method sections or elsewhere, N = statistical section is lacking, [calculated], #untreated *Npc*-/- were euthanized at end-stage of the disease at 9 weeks of age

**Table 3:** Experimental groups

References	Experimental groups						
	Wild-type		Untreated *Npc*-/- mice		Treated *Npc*-/- mice		Total
	Females	Males	Females	Males	Females	Males	
[16]	9		*Npc1*^*nih*^: 6 *Npc1*^*nmf164*^: 3		*Npc1*^*nih*^ AAV9-GAPDH: 6 AAV9-PGK: 6 AAV9-NPC1: 6 AAV9-CBA: 6 AAV9-SYN: 6 AAV9-SYN-S: 6 AAV9-SYN-D: 6 AAV9-CBA: 6 AAV9-CAG: 6 *Npc1*^*nmf164*^ AAV9-SYN: 3 AAV9-NPC1: 3		78
[11]	9 weeks: 3 End-stage: 5	9 weeks: 2 End-stage: 3	9 weeks: 1 End-stage: 6	9 weeks: 4 End-stage: 2	9 weeks AAV9: 4 AAV-PHP.B: 2 End-stage: AAV9: 4 AAV-PHP.B: 5	9 weeks AAV9: 3 AAV-PHP.B: 2 End-stage: AAV9: 5 AAV-PHP.B: 4	55
[22]	n.s.		8	10	8	10	36
[12]	11		Saline-treated, gel-food: 11 Untreated, gel-food:6 Untreated, standard food: 6		12		46
[15]	6		8		Low dose: 8 High dose: 8 Miglustat: 8		38
[14]	14–29	11–21	6–13	5–12	3–11	6–14	n.s.
[13]	5		7–17		8–16		n.s.
[10]	0		Untreated: 16 AAV9-GFP: 6		Neonates: AAV9-CamKII: 6 Juvenile AAV9-CamKII: 9 AAV9-EF1α: 7		44

Mice used for investigating the biodistribution of the vector are not included in the experimental groups. *n.s.* not stated

## Inclusion and exclusion criteria

Expectedly, the number of animals in the NPC studies changes at different time points since *Npc-/-* mice are reaching the end stage of the disease as the study progresses. Exclusion criteria can in this case reflect humane endpoints where animals are euthanized before the predetermined endpoints [20]. Six out of the eight studies had included humane endpoints, whereas, in the remaining two studies, mice were allowed to die due to severe neurological symptoms consequently making the mice unable to, e.g., eat solid food [12, 13]. When assessing the efficacy of the gene therapy strategy, survival is an important parameter to measure. Still, it can be challenging to compare the efficiency of the viral vectors when different humane endpoints were implemented, thus different criteria for euthanasia were used, or when no humane endpoints were used at all (Table 4).

**Table 4:** Humane endpoints

Parameter	Observation	References
Weight loss	15% loss of body weight after a 24 h period	[16]
	30% weight loss of maximum weight	[11]
	> 15% loss of body weight in a week	[14]
	Loss of 1 g of body weight within a 24 h period	[15]
	Rapid weight loss	[10]
Locomotor function	Reluctance to move, repeated falling to the side during forward ambulation	[11]
	Tremor, abnormal gait, unbalanced	[14, 22]
	Severe loss of motor function	[10]
Skin and fur	Ruffled fur, yellow coat	[22]
Eyes	Palpebral closure/eyes appearing dull rather than bright	[11]
General appearance	Penile prolapse	[14]

Another important aspect when reporting exclusion criteria is to state whether any animals or data points were excluded from the analysis and why. Only one study reported whether any inclusion or exclusion criteria were set or whether any animals or data points were excluded from the study [11]. In most of the studies, there was an inconsistency in the number used in a specific analysis compared to the total number of mice in the experimental group. For each analysis, the number of animals in each group can differ from the total number of animals used. It is, therefore, important to report the exact value of the number of animals in each experimental group for every analysis performed. For most outcome measures, the number of mice per experimental group is clearly stated, e.g., in figure legends. Still, there is an inconsistency when reporting the number of animals in immunohistochemical evaluation where the number of mice is rarely reported. As stated in the ARRIVE guidelines, when measurements are collected at different time points, a full description of which animals undergo measurement and when should be reported [20]. Clearly stating the actual number of animals used for testing the particular hypothesis allows for transparency and gives the reader an idea of the value of the specific result.

## Randomization and blinding

Even though the ARRIVE guidelines have been accessible for more than 10 years, details of, e.g., randomization and blinding are still missing from most publications [20, 24, 25], and this was also evident when reviewing the included papers (Table 3). Only one out of eight studies provided details on both randomization and blinding strategy, although the details concerning randomization were limited [11]. Davidson and colleagues argue that they have employed randomization by using multiple cohorts, but this does not depict the type of randomization used to allocate the mice to either the control or treatment group (e.g., simple, stratified, or block randomization) [26]. On the other hand, the authors state that they minimized confounders by including mice within each treatment group in every cohort, which complies with the ARRIVE guidelines. Besides the aforementioned study, only one other study included information about blinding [10]. These findings are worrisome, based on the knowledge that randomization and blinding reduce bias in animal research [24, 27, 28].

## Outcome measures

The ARRIVE guidelines clearly state: “define all outcome measures assessed” [20]. The outcome measures refer to those variables recorded during an experiment, e.g., used to assess the effect of an intervention. Therefore, it is important to report all outcome measures assessed, otherwise, there is a risk of only including statistically significant data [20].

The pathological hallmarks of the disease in *Npc-/-* mice are progressive weight loss, hepatosplenomegaly, and neurodegeneration resulting in a fatal outcome of the disease [29–31]. In addition, the cerebellum is severely affected and presents with Purkinje cell loss, reactive microglia, and astrogliosis [14, 32]. These parameters are, therefore, also the primary focus when evaluating the efficiency of gene therapy in mice models of NPC disease. One of the first visible symptoms in NPC-deficient mice is weight loss, and this parameter is also evaluated in all studies [10–12, 14–16, 22] except one [13] (Table 5). All studies testing the effect of an AAV vector showed a delay in the onset of weight loss. Another important outcome measure assessed is survival, which was also evaluated in all included studies (Table 6). However, there are several important issues to consider when comparing NPC disease studies. Also noted by Hughes and colleagues, differences in vector serotype, vector dose, timing and route of administration, transgene cassettes, and humane endpoints make direct comparison between studies almost impossible [15].

**Table 5:** Body weight

Peak BW untreated *Npc*-/-	Peak BW treated *Npc*-/-	Vector	Administration	References
n.a.*				[16]
6 weeks	14 weeks	AAV-PHP	Systemic	[11]
	8 weeks	AAV9		
7 weeks	15 weeks	AAV9/3	CNS	[12]
6 weeks	12 weeks	AAV9 low dose	CNS	[15]
	16 weeks	AAV9 high dose		
8 weeks	18 weeks (f)	AAVrh.10	CNS	[14]
	16 weeks (m)			
n.a.				[13]
6 weeks	8 weeks	AAV9-CamKII	Systemic	[10]
	12 weeks	AAV9-EF1α		
6 weeks	6 weeks	TfRMAb targeted THLs	Systemic	[22]

*BW* bodyweight, *f* females, *m* males, *n.a.* not assessed, *peak body weight is not assessed, *CNS* central nervous system

**Table 6:** Survival

Median survival untreated *Npc*-/-	Median survival treated *Npc*-/-	Lifespan extension (%)	Vector	Longest survival	References
76 days	263 days	246	AAV-NPC1	275 days	[16]
72 days	234 days	225	AAV-PHP	> 1 year (AAV-PHP)	[11]
	112 days	56	AAV9		
75 days	205 days	173	AAV9/3	310 days	[12]
75 days	116.5 days	55	AAV9 low dose	126 days	[15]
	158 days	111	AAV9 high dose	168 days	
112 days (f)	280 days (f)	150	AAVrh.10	280 days (f)	[14]
98 days (m)	168 days (m)	71			
71 days	94 days	32	AAV9	100 days	[13]
69 days	103 days (j)	49	AAV9-CamKII	140 days (j)	[10]
	166 days	141	AAV9-EF1α	300 days	
75 days*	75 days	n.s.	TfRMAb targeted THLs	77 days	[22]

*f* = females, *m* = males (euthanized due to the development of penile prolapse, starting at 24 weeks of age), *j* = juvenile at the time of injection, * = data are reported as mean, n.s. = non-significant

The involvement of the cerebellum with progressive Purkinje cell loss in NPC disease becomes evident when the patient develops symptoms such as tremors and ataxia, which are also evident in Npc-/- mouse models [1, 33–36]. Furthermore, these severe symptoms affect the ability to accomplish normal behavioral tasks involving motor activity [14, 37]. Assessment of motor coordination is therefore often included when evaluating gene therapy in Npc-/- studies, and six out of eight studies included in this review, have also assessed motor function using, e.g., the balance beam, open field activity, rotarod, and/or gait analysis (Table 7). Lastly, a broad spectrum of post-mortem parameters was included in the outcome measures, with the main focus on neuropathology. Purkinje cell degeneration is the most commonly evaluated parameter using the Purkinje cell marker Calbindin [38] and seven out of eight papers have analyzed Purkinje cell degeneration in the Npc-/- mouse model (Table 7). As mentioned earlier, Purkinje cell loss is a hallmark of the NPC disease, and the development of symptoms is related to the extent of Purkinje cell degeneration. Thus improvement in lifespan is usually accompanied by the preservation of these vulnerable cerebellar neurons [10, 39]. The information found in Table 7 can provide an overview of different methods to be included in future studies on NPC disease.

**Table 7:** Outcome measures

Outcome measures		References
Behavioral analysis		
Motor coordination	Rotarod	[12, 13]
	Gait analysis^*^	[15, 14, 11, 12, 16]
	Balance beam	[14, 11]
Locomotor activity	Open field	[13, 15, 14]
Tremor	Automated tremor sensor	[16]
Brain pathology		
Purkinje cell degeneration	Calbindin staining	[10, 13, 15, 14, 12, 16]
	Nissl staining	[15]
	H&E	[13, 22]
Cholesterol accumulation	Filipin staining	[10, 15, 14, 11]
Lipid accumulation	GM2 staining	[11]
Glycogen accumulation	PAS staining	[14]
Neuroinflammation	Astrogliosis (GFAP)	[15, 14, 22, 11, 16]
	Microglia (CD68, Iba-1)	[15, 14, 11, 16]
Liver pathology		
	H&E	[14, 22, 11]
Inflammation	CD68	[10, 11]
Cholesterol accumulation	Filipin	[10, 15, 11, 12]
Spleen pathology		
	H&E	[14, 22]
Cholesterol accumulation	Filipin	[15, 22]

^*^Gait analysis included automated gait analysis using the Noldus CatWalk [15, 16], visual inspection of footprints [14], clinical description [12], and gait scoring scheme [11]

It has been proposed that registering a protocol in advance, as seen with clinical trials, can avoid data dredging also called p-hacking. This means selective reporting of outcome measures that were statistically significant or collecting more data afterward in a search for significant results [20, 40–42]. However, this calls for a general change in the field of research where publication bias is a major challenge. There is evidence that studies with positive or statistically significant (*p* < 0.05) results are more likely to get published, and that neutral or negative findings take longer to publish, are published in low impact, or are not published at all [41, 43]. This is indeed worrisome as publication bias can lead to overestimation of, e.g., treatment effects [41], which undoubtedly further challenges the translation of results from animal studies to humans.

## Statistical methods

To fulfill the requirements stated by the ARRIVE guidelines, details of the statistical methods used as well as statistical software should be provided. Furthermore, whether the data met the assumptions for the statistical approach should also be described [20]. Based on these criteria, only one of the included papers fulfills these requirements [11]. Two of the papers did not include a section about the statistical methods used in the study [14, 22]. The remaining papers do not provide sufficient details about the statistical methods, and none of these papers provided detail about the assumptions of the underlying data making it difficult for the reader to assess the appropriateness and suitability of the methods used. Assumptions for using parametric tests are, e.g., that the data analyzed are continuous, follow a normal distribution, and the variances between different groups are similar [20]. Usually, parametric tests have higher statistical power compared to non-parametric tests [19, 44]. Nevertheless, they require that the aforementioned assumptions are met. The use of incorrect assumptions can result in false positive results, consequently making invalid conclusions. Inappropriate reporting of statistical methodology in combination with the aforementioned experimental bias is a common cause of poor study design [45].

## Experimental animals

Species-appropriate details should be provided including species, strain, sex, age, weight, the origin of the animals, health status, genetic modification, and genotype [20].

### Mouse models for NPC

Most NPC disease studies to date use the BALB/cNctr-*NPC1*^*m1N*^*/J* (*Npc*^*nih*^) murine model, which was also evident when reviewing the literature about gene therapy as a treatment strategy in *Npc1-/-* mice. Six out of eight studies included in this review used the *Npc*^*nih*^, whereas only one used the FVB.C-*NPC1*^*m1N/J*^ mouse model. One study included both *Npc*^*nih*^ and *Npc*^*nmf164*^ mice to test the effect of the gene therapy [16]. Only one study to date has evaluated viral gene therapy in *Npc2*-/- mice [14]. All included studies provide sufficient data on the species, strain, genotype, genetic modification, the origin of the animals as well as the age of the mice at the beginning of the study, which complies with the ARRIVE guidelines. A single study provided details on health status [13]. An important detail when reporting animal studies is the sex of the species used. In a lot of different research areas, sex-dependent differences related to lifespan, immune activation, and response to therapy have been observed [46–49], which are also evident in studies using NPC-deficient mice [8, 14, 50, 51]. The NPC disease phenotype is, e.g., more severe in *Npc*-/- male mice with shorter survival, more dramatic weight loss, and a more progressive Purkinje cell degeneration compared to female *Npc-/-* mice [37, 52, 53]. Despite these well-known differences, the sex of the animals was only specified in three of the eight articles (Table 3). Sex and species-specific parameters such as strain and age contribute to variance between studies [42]. Therefore, when certain details, such as sex, are not reported, it may pose challenges to the reproducibility and translatability of the study.

## Experimental procedures

The reporting of the experimental procedures should be described in enough detail to make it possible for other researchers to replicate them [20]. For all studies included, the AAV subtypes, including promotor, vehicle, doses, volumes, and route of administration were described in detail (Table 1), as well as the rationale for these decisions. However, the specific procedure for the administration of viral vectors and euthanasia lacks several details. Stereotaxic surgery was used in three of the included studies [12, 14, 15], and a thorough description of the surgical procedure would therefore be expected. According to the ARRIVE guidelines, information about surgery includes a specific description of the procedure (incl. sham surgery), anesthetics used (drug, dose, concentration, route of administration), analgesia (pre-and post), aseptic techniques, and parameters monitored during anesthesia (e.g., assessment of the surgical anesthetic plane), duration of the procedure and anesthesia. All these aspects are another essential part of the previously mentioned 3Rs, specifically refinement. In one of the studies stereotaxic surgeries were performed in mice at 6 weeks of age [14]. The only information provided was the anesthesia used (3% isoflurane) and the injection volume of the viral vector. Unfortunately, e.g., analgesia regimen was not mentioned, although this is an important part of securing animal welfare in research. Two studies used retro-orbital injections in weanlings as the route of vector administration, which requires anesthesia [54], but information about anesthesia is lacking [10, 11]. The same limitations were seen when reporting the use of euthanasia. Four out of eight studies did not provide any details of the anesthetics used and/or the procedure used for euthanasia [13, 15, 16, 22]. Although minimizing pain and distress in laboratory animals is the consensus when performing animal studies, the type and use of analgesics during experimental surgery in rodents are often not reported [55], which was unfortunately also the case in the papers included in this review.

## Results

As for the statistical methods, the reporting of results, including descriptive statistics for each experimental group (e.g., mean and standard deviation (SD)) [20], are also limited or inconsequent in the reviewed studies. One of the included studies has not provided the reader with any statistical information about the results, neither in the text nor in the figure legends [12]. Four of the included studies use SD and standard error of the mean (SEM) interchangeably [10, 14, 15, 22]. Importantly, SEM provides information about how accurate the sample mean is and whether the mean is a precise estimate of the population mean. SD gives information about the variability within the group. Thus, data should be summarized using SD [56].

Furthermore, SD is used for calculating the effect size, the last parameter that should be reported according to the ARRIVE guidelines. None of the studies has included information about the effect size, which unfortunately often is the case in animal research [20]. Nevertheless, the effect size together with its confidence interval is an important part of the statistical information providing the reader with information about the estimated magnitude of differences between the experimental groups, the precision of the estimate, as well as whether the findings are biologically relevant [57].

## Summary

At the moment, thousands of journals are recommending the ARRIVE guidelines [20], and more will definitely follow. However, there are still some challenges we need to overcome. Three of the included papers refer specifically to ARRIVE guidelines, and state that the study design is based on the ARRIVE guidelines 2.0 [11], or that the animal studies were conducted according to the ARRIVE guidelines and recommendations [15, 16]. None of the remaining papers provide such information.

Interestingly, four journals clearly state that animal studies should comply with ARRIVE guidelines [10, 14–16, 22]. Two of these journals explicitly state that when preparing the manuscript, the ARRIVE guidelines must be followed [10, 15, 16], whereas the two other journals report that the author must state or confirm that the experiments comply with the ARRIVE guidelines, e.g., in the method Sect. [14, 22]. Two other journals encourage following the ARRIVE guidelines [11, 13]. In one journal, no information is provided in the submission guidelines [12]. It is, however, unknown when this editorial information was last updated. More responsibility from the journals to ensure the needed information is reported is probably needed, which has also been emphasized by, e.g., The Journal of Bone and Joint Surgery with the publication of the editorials; “JBJS Will Require Adherence to ARRIVE Guidelines for Animal Research to Reduce Bias and Improve Quality of Reporting” [58]. In this editorial, they highlight that all manuscripts reporting animal studies will include an annotated checklist of the ARRIVE guidelines from the 1st of January 2020. Furthermore, they recognize the importance of their role as editors, e.g., concerning the problem of publication bias, and will encourage the publication of both positive and negative results [58]. Even though several journals endorse these guidelines, essential information is still lacking from scientific publications [59], which also was the case in the papers included in this review. Surprisingly, in a survey of Swiss researchers, more than half of the authors whose last publication was in a journal endorsing the ARRIVE guidelines had never heard of these guidelines before [60]. The majority of the researchers included in the survey were engaged in biomedical or medical research [60]. In addition, fulfilling a checklist at submission was not sufficient to adhere to the ARRIVE guidelines [61]. More awareness is needed, and this requires first and foremost adoption of these guidelines by the journals [58], but also an acceptance from researchers of why these guidelines are of importance and the risks associated with introducing bias in animal research when the guidelines are not followed [20, 60].

## Conclusions

Even though the first ARRIVE guidelines were published more than 10 years ago, the reporting of animal research is still not following these guidelines. This is unfortunately not only the case for studies in NPC disease but for many other research areas using animals to study, e.g., cancer, stroke, infectious, and cardiovascular diseases [17, 21, 24, 62–65].

Animal models are essential for developing new treatment strategies for incurable diseases and learning more about the pathogenesis of NPC disease. During the last twenty years, the research in the field of NPC disease has increased considerably, even though a cure for the disease is still lacking [8].

When reviewing and evaluating whether the papers (all published from 2017 and forth) were published in compliance with the ARRIVE guidelines, none fulfilled the minimum required information to be included when reporting animal research. Most of the papers lacked information in several important areas including justification of sample size, blinding, randomization, and statistical methodology. Without this information, readers and reviewers cannot assess the reliability of the findings. This emphasizes the need for more comprehensive reporting in animal research and that detailed descriptions of the experimental procedures and animal models must be available to ensure that results can be evaluated, interpreted, and replicated in the future.

Despite these guidelines being recommended by more than a thousand journals [20], there is still a lack of adherence to the ARRIVE guidelines in several research areas [66]. The successful implementation of the ARRIVE guidelines requires increased awareness from the entire scientific community. With the publication of ARRIVE 2.0 in 2020, where they have highlighted “the essential 10” to help scientists, editors, and reviewers focus on the minimum information needed to be reported when publishing pre-clinical studies [20], future research can hopefully become more transparent. Furthermore, the guidelines can be helpful for the researcher during the planning and conducting of animal studies, ensuring rigorous study design and collecting sufficient information for the preparation of the manuscript [20].

Raising more awareness of the ARRIVE guidelines and the importance of reporting adequate information on study design, including randomization and blinding, in combination with conducting systematic reviews, can hopefully contribute to solving the translational and reproducibility challenges in preclinical research in the future, as also stated by Alstrup and Sonne [17].

One step at a time, but when “the essential 10” is fully implemented in the backbone of animal research, all 21 items included in the ARRIVE guidelines can hopefully also be implemented.

## Data Availability

Data sharing is not applicable as no new data were created or analyzed in this review.

## Abbreviations

AAV: Adeno-associated virus
ARRIVE: Animal research: reporting of In vivo experiments
GFP: Green fluorescent protein
NPC: Niemann-Pick type C
PBS: Phosphate-buffered saline
SD: Standard deviation
SEM: Standard error of the mean
