# Correction: Reporting preclinical gene therapy studies in the field of Niemann-Pick type C disease according to the ARRIVE guidelines

**DOI:** 10.1186/s13023-025-03826-w

**Published:** 2025-06-10

**Authors:** Charlotte Laurfelt Munch Rasmussen, Annette Burkhart, Torben Moos, Louiza Bohn Thomsen

**Affiliations:** 1 Neurobiology Research and Drug Delivery, Department of Health Science and Technology, Aalborg University, Selma Lagerløfs Vej 249, DK-9260 Gistrup, Denmark; 2 The Biomedical Laboratory, Department of Molecular Medicine, University of Southern Denmark, J.B. Winsløws Vej 23, DK-5000 Odense C, Denmark

**Correction: Orphanet Journal of Rare Diseases (2025) 20:214** 10.1186/s13023-024-03479-1

Following publication of the original article [1], the authors reported that at the end of the section “Outcome measures,” a paragraph is absent. Furthermore, Table 7 is missing.

**Table 7:** Outcome measures

	Outcome measures	References
*Behavioral analysis*		
Motor coordination	Rotarod	[12, 13]
	Gait analysis^*^	[11, 12, 14–16]
	Balance beam	[11, 14]
Locomotor activity	Open field	[13–15]
Tremor	Automated tremor sensor	[16]
*Brain pathology*		
Purkinje cell degeneration	Calbindin staining	[10, 12–16]
	Nissl staining	[15]
	H&E	[13, 22]
Cholesterol accumulation	Filipin staining	[10, 11, 14, 15]
Lipid accumulation	GM2 staining	[11]
Glycogen accumulation	PAS staining	[14]
Neuroinflammation	Astrogliosis (GFAP)	[11, 14–16, 22]
	Microglia (CD68, Iba-1)	[11, 14–16]
*Liver pathology*		
	H&E	[11, 14, 22]
Inflammation	CD68	[10, 11]
Cholesterol accumulation	Filipin	[10, 11, 12, 15]
*Spleen pathology*		
	H&E	[14, 22]
Cholesterol accumulation	Filipin	[15, 22]

*Gait analysis included automated gait analysis using the Noldus CatWalk [15, 16] visual inspection of footprints [14], clinical description [12], and gait scoring scheme [11]

The paragraph and Table 7 have now been added and are the following:

“The involvement of the cerebellum with progressive Purkinje cell loss in NPC disease becomes evident when the patient develops symptoms such as tremors and ataxia, which are also evident in Npc-/- mouse models [1, 33–36]. Furthermore, these severe symptoms affect the ability to accomplish normal behavioral tasks involving motor activity [14, 37]. Assessment of motor coordination is therefore often included when evaluating gene therapy in Npc-/- studies, and six out of eight studies included in this review, have also assessed motor function using, e.g., the balance beam, open field activity, rotarod, and/or gait analysis (Table 7). Lastly, a broad spectrum of post-mortem parameters was included in the outcome measures, with the main focus on neuropathology. Purkinje cell degeneration is the most commonly evaluated parameter using the Purkinje cell marker Calbindin [38] and seven out of eight papers have analyzed Purkinje cell degeneration in the Npc-/- mouse model (Table 7). As mentioned earlier, Purkinje cell loss is a hallmark of the NPC disease, and the development of symptoms is related to the extent of Purkinje cell degeneration. Thus improvement in lifespan is usually accompanied by the preservation of these vulnerable cerebellar neurons [10, 39]. The information found in Table 7 can provide an overview of different methods to be included in future studies on NPC disease.

It has been proposed that registering a protocol in advance, as seen with clinical trials, can avoid data dredging also called p-hacking. This means selective reporting of outcome measures that were statistically significant or collecting more data afterward in a search for significant results [20, 40–42]. However, this calls for a general change in the field of research where publication bias is a major challenge. There is evidence that studies with positive or statistically significant (*p* < 0.05) results are more likely to get published, and that neutral or negative findings take longer to publish, are published in low impact, or are not published at all [41, 43]. This is indeed worrisome as publication bias can lead to overestimation of, e.g., treatment effects [41], which undoubtedly further challenges the translation of results from animal studies to humans.”

The original article [1] has been updated.
